# Role of Device-Assisted Enteroscopy in Crohn’s Disease

**DOI:** 10.3390/jcm13133919

**Published:** 2024-07-04

**Authors:** Giulia Catassi, Clelia Marmo, Antonio Gasbarrini, Maria Elena Riccioni

**Affiliations:** 1 Digestive Endoscopy Unit, IRCCS “Agostino Gemelli” University Hospital, Catholic University of Rome, Largo A. Gemelli 8, 00168 Rome, Italy; clelia.marmo@outlook.it; 2 Pediatric Gastroenterology and Liver Unit, Umberto I Hospital, Sapienza University of Rome, Largo A. Gemelli 8, 00168 Rome, Italy; 3 Internal Medicine, Gastroenterology and Liver Unit, “Agostino Gemelli” University Hospital, Catholic University of Rome, Largo A. Gemelli 8, 00168 Rome, Italy; antonio.gasbarrini@policlinicogemelli.it

**Keywords:** enteroscopy, Crohn’s Disease

## Abstract

Crohn’s Disease (CD) is a chronic inflammatory disorder of the gastrointestinal tract, posing diagnostic and management challenges due to its potential involvement of any segment from the mouth to the anus. Device-assisted enteroscopy (DAE) has emerged as a significant advancement in the management of CD, particularly for its ability to access the small intestine—a region difficult to evaluate with conventional endoscopic methods. This review discusses the pivotal role of DAE in the nuanced management of CD, emphasizing its enhanced diagnostic precision and therapeutic efficacy. DAE techniques, including double-balloon enteroscopy (DBE), single-balloon enteroscopy (SBE), and the now-withdrawn spiral enteroscopy, enable comprehensive mucosal assessment, targeted biopsies, and therapeutic interventions like stricture dilation, bleeding control, and foreign body removal. Despite its benefits, DAE carries risks such as perforation, bleeding, and pancreatitis, which require careful procedural planning and a skilled execution. The review highlights DAE’s impact on reducing surgical interventions and improving patient outcomes through minimally invasive approaches, thereby enhancing the quality of life for patients with CD. Continuous improvement and research are essential in order to maximize DAE’s utility and safety in clinical practice.

## 1. Introduction

Crohn’s Disease (CD) is a chronic, inflammatory condition of the gastrointestinal tract, classified under the umbrella of inflammatory bowel diseases (IBDs). The etiology of CD is complex, involving an interplay of genetic factors, environmental triggers, immune system responses, and microbiota composition [1,2]. CD is distinguished by its potential to affect any part of the gastrointestinal tract, from the mouth to the anus, and presents a discontinuous, segmental distribution, which complicates both diagnosis and management [3]. Small-bowel (SB) lesions are recognized in 30 to 60% of CD patients, and 10% to 30% of individuals have isolated SB disease [4]. Because these lesions cannot be recognized with upper and lower endoscopy alone—the conventional endoscopic methods—isolated SB CD is challenging to identify [4]. The development of device-assisted enteroscopy (DAE) techniques in the early 21st century have substantially impacted the diagnosis and treatment of Crohn’s, particularly with its capacity to access and assess the small intestine, a region elusive to conventional endoscopic methods, such as push enteroscopy and capsule endoscopy [5].

The evolution of DAE techniques marks a significant milestone, shifting from limited traditional methods to enabling direct visualization and intervention across the entire small bowel. This advancement has been crucial for a more accurate and comprehensive diagnosis and treatment of small-bowel diseases, particularly CD, where direct mucosal evaluation, targeted biopsies, and therapeutic interventions like stricture dilation, bleeding control, and foreign bodies removal are now possible [6].

## 2. Purpose of the Review

The primary aim of this review is to discuss the substantial role DAE plays in the nuanced management of CD. It will examine the enhanced diagnostic precision DAE brings to the table, as well as its therapeutic efficacy in managing this intricate disease. The review will highlight DAE’s ability to visualize the entire small intestine, which is frequently involved in CD; facilitate targeted biopsies and thorough mucosal assessments; and identify complications such as strictures, fistulas, and areas of active inflammation. Moreover, the review will emphasize the impact of DAE on therapeutic interventions, from the dilation of strictures to the control of bleeding, removal of foreign bodies, and application of local therapy to inflammatory lesions. By detailing these aspects, the review intends to demonstrate the integral role DAE plays in offering an advanced approach to CD management, potentially leading to enhanced patient outcomes and an improved quality of life.

## 3. Procedure Overview and Technique

DAE has significantly evolved, with various techniques offering unique benefits and limitations in the management of small-bowel diseases, including Crohn’s Disease. DAE encompasses double-balloon enteroscopy (DBE), single-balloon enteroscopy (SBE), and spiral enteroscopy, each offering unique access to the small intestine [7].

### 3.1. Double-Balloon Enteroscopy (DBE)

The DBE technique uses two balloons, one on the endoscope and another on a flexible overtube, which alternately inflate and deflate to pleat the small intestine over the overtube and endoscope, allowing a deep traversal of the small bowel [8]. Despite its utility, DBE has limitations, particularly in terms of the extent of the bowel that can be examined in a single session [9]. A complete small-bowel enteroscopy with DBE is time-consuming and often requires full anesthesia, making it less practical for extensive examinations [10]. However, DBE is particularly advantageous for patients with prior surgeries, as it allows for the easier navigation through postoperative adhesions and altered anatomy compared to spiral enteroscopy techniques [11].

### 3.2. Single-Balloon Enteroscopy (SBE)

SBE uses a single balloon on an overtube to assist in guiding the endoscope through the small intestine by advancing and stabilizing the overtube sequentially [12]. While SBE offers a simpler setup compared to DBE, it may not achieve the same depth of insertion [13]. However, SBE has proven effective for both diagnostic and therapeutic purposes and is often preferred for its relative ease of use and shorter procedure times. SBE is advantageous in patients where full anesthesia might be a concern, as it can often be performed under deep sedation [14]. There is also a modality of SBE where the balloon is inserted through the scope itself, eliminating the need for an overtube. This approach involves a balloon catheter that can be passed through the working channel of the endoscope, which is then inflated to help advance and stabilize the endoscope. This method simplifies the procedure further and can reduce the time required for setup and execution [15,16].

### 3.3. Spiral Enteroscopy (SE)

SE employs a spiral-shaped overtube that, when rotated, pleats the small intestine onto the overtube, propelling the endoscope forward [17]. Initially, SE was “manual”, meaning manually rotated by the endoscopist. It was particularly advantageous for patients with upper intestinal polyps. The spiral-shaped overtube allowed the endoscope to be withdrawn through it, facilitating the easy harvesting of polyps. This feature provided a distinct advantage over other enteroscopy techniques. Then, recent developments have introduced motorized spiral enteroscopy (MSE). which represents an evolution of the manual spiral technique, incorporating a motorized system to automate the rotation of the spiral overtube. The motorized system offers several advantages over its manual predecessor, including a more controlled and consistent rotation, a reduced physical strain on the endoscopist, and potentially shorter procedure times. MSE uses an electric motor to rotate the overtube, allowing for the precise and continuous advancement of the endoscope through the small intestine. Studies have demonstrated its effectiveness in reaching deep segments of the small bowel, making it a valuable tool in the management of Crohn’s Disease (CD) [12,18]. However, SE has been withdrawn from the market in July 2023 due to severe adverse events [19].

DAE is commonly performed under deep sedation or general anesthesia to ensure patient comfort and facilitate a thorough examination. X-ray surveillance, typically fluoroscopy, is often used during DAE to guide the procedure, particularly in complex cases where navigation through the small intestine is challenging [20]. Fluoroscopy can help in accurately positioning the endoscope and overtube, especially when dealing with anatomical variations or postoperative adhesions. However, the necessity of X-ray surveillance is not absolute for all DAE procedures [21]. In many cases, experienced endoscopists can perform DAE without the need for continuous fluoroscopic guidance, relying instead on anatomical landmarks and tactile feedback to navigate the small intestine. This approach can reduce radiation exposure to both the patient and the medical team [21] The decision to use X-ray surveillance depends on various factors, including the complexity of the case, the experience of the endoscopist, and the specific clinical scenario [21].

The choice between DBE and SBE depends on clinical indications, operator expertise, and device availability [22]. The procedural approach, anterograde (oral) or retrograde (anal), is chosen based on the small-intestine segment requiring examination, as suggested by symptoms and imaging, or WCE [23]. Certain symptoms and clinical presentations can help localize the disease burden within the small intestine, thus informing the choice of approach: patients with jejunal involvement often present with symptoms such as upper abdominal pain, bloating, and early satiety; ileal involvement is commonly associated with symptoms such as lower abdominal pain, cramping, diarrhea, and sometimes blood in the stool [24,25]. In some cases, if the exact location of the disease is unclear, imaging can be performed first. For patients without suspicion or evidence of stenoses, it is possible to precede DAE with a capsule endoscopy to identify the level or area of interest. If the area of interest is within the first 75% of the small-bowel transit time, an oral approach is recommended. Conversely, if it is beyond 75%, an anal approach is indicated. This strategy is particularly useful when addressing small-intestinal ulcers and bleeding [26]. 

The duration of DAE is variable, depending on the complexity of the case and the extent of the small bowel that needs to be examined [27]. Following the procedure, patients typically undergo a recovery phase to offset the effects of sedation or anesthesia. The majority of patients are discharged on the same day, provided there are no complications or need for extended observation. Post-procedural care includes dietary advice and monitoring for signs of potential complications such as abdominal pain, fever, or bleeding, which require prompt medical attention [28]. For dietary advice, there are no specific guidelines; however, the same dietary recommendations as those used post-procedure are followed: immediately after the procedure, patients are advised to start with clear liquids such as water, clear broths, or tea. If clear liquids are well-tolerated, patients can gradually progress to a soft diet within the first 24 h. Soft foods include mashed potatoes, yogurt, applesauce, and well-cooked vegetables. These dietary guidelines can help minimize the risk of post-procedural complications and improve patient outcomes.

The patients eligible for DAE are as follows:

- Patients who have undergone an endoscopy with negative results but have indications of Crohn’s Disease (CD) based on MRI or small-bowel capsule endoscopy findings—device-assisted enteroscopy can be utilized for endoscopic and histological confirmation of the diagnosis [2];
- When clinical symptoms suggest small-bowel involvement that remains unexplained after initial non-invasive investigations [2];
- When therapeutic maneuvers such as stricture dilation, control of bleeding, or removal of foreign bodies are needed [2].

In these scenarios, DAE serves as a strategic choice to bridge the gap between initial non-invasive imaging and the need for a more definitive diagnosis and therapeutic intervention [29].

## 4. Advantages of Device-Assisted Enteroscopy (DAE) over Other Methods

DAE has shown numerous advantages over other diagnostic methods in Crohn’s Disease, such as magnetic resonance enterography (MRE), computed tomography enterography (CTE), and wireless capsule endoscopy (WCE) [30]. It should be considered complementary to a non-invasive examination of the small intestine, as it provides not only a direct and detailed visualization of the mucosal surface (with a sensitivity for detecting small-bowel lesions in Crohn’s Disease of 65%), but also the unique opportunity for biopsy and therapeutic interventions [31]. While MRE and CTE are critical for a comprehensive structural assessment and initial suspicion of Crohn disease, they cannot offer a direct mucosal assessment and histological examination [32]. WCE allows for a broader visualization of the mucosal surface and is sensitive for detecting small-bowel lesions, but it has limitations due to the inability to take biopsies, its diagnostic-only capacity, and the risk of capsule retention [33].

MRE, in particular, is a non-invasive sectional imaging modality that is highly useful for following up on inflammatory activity. It can detect fistulas or strictures with a high sensitivity and specificity, making it an excellent tool for monitoring disease progression and complications. The sensitivity of MRE for detecting small-bowel active inflammation in Crohn’s Disease is approximately 68%, with a specificity of approximately 95% [34]. These rates highlight MRE’s effectiveness in non-invasively assessing disease activity and complications. Moreover, MRE also serves as a valuable prognostic tool in Crohn’s Disease. The Magnetic Resonance Index of Activity (MaRIA) score is a validated scoring system used in MRE to quantify disease activity: it incorporates parameters such as bowel wall thickness, edema, ulceration, and contrast enhancement, providing an objective measure of inflammatory activity in Crohn’s Disease. Studies have shown that the MaRIA score correlates well with endoscopic findings and can predict the risk of surgery, making it an effective tool for guiding treatment strategies and monitoring disease progression over time [35].

Despite these strengths, MRE and CTE lack the capability for a direct mucosal visualization and histological examination. This limitation is where DAE excels, offering not just a detailed visualization but also the ability to perform therapeutic interventions. This is especially significant in Crohn’s Disease management, where the accurate assessment of the disease extent, activity, and complications can directly influence treatment decisions and patient outcomes. DAE allows for the biopsy of suspicious areas, dilation of strictures, and treatment of bleeding lesions, providing a comprehensive approach to disease management that cannot be matched by non-invasive imaging techniques alone [36].

## 5. Role of DAE in Diagnosing Crohn’s Disease

The role of DAE in diagnosing Crohn’s Disease is multifaceted, offering significant advantages over traditional diagnostic methods. First, it is unparalleled in its ability to visually access the entire small intestine, providing a favorable diagnostic yield of up to 80%, with a low complication rate which underscores its safety [37,38,39]. It, indeed, enables the detection of early mucosal changes that are indicative of Crohn’s Disease and are often missed by other diagnostics, including minor erosions, aphthous ulcers, or early inflammatory lesions [40]. The ability to perform targeted biopsies is instrumental in confirming the diagnosis, as the histopathological examination of biopsy samples can reveal granulomas or other microscopic features characteristic of CD, thus enhancing diagnostic precision [41]. Moreover, DAE can predict the risk of surgery in Crohn’s Disease patients, with the small-bowel simple endoscopic score for Crohn’s Disease (SES-CD) serving as a key prognostic tool, thus influencing treatment decisions and patient management strategies. Research carried out at Samsung Medical Center discovered a significant rise in the likelihood of surgical complications in patients with a small-bowel simple endoscopic score for Crohn’s Disease (SES-CD) of 7, as opposed to those with a small-bowel SES-CD of 6 [42]. 

While DAE provides a high diagnostic yield, there are instances where the procedure may not be able to visualize the entire small intestine. In such cases, alternative or complementary diagnostic methods (imaging or WCE) can be employed to achieve a comprehensive evaluation.

## 6. Therapeutic Applications of DAE in Crohn’s Disease

DAE not only plays a pivotal role in the diagnosis of Crohn’s Disease but also offers a range of therapeutic applications such as balloon stricture dilation, steroid injections, the treatment of bleeding ulcers, and the removal of foreign bodies. These interventions can directly address complications associated with the disease, potentially reducing the need for surgical interventions and improving patient outcomes [43].

### 6.1. Stricture Dilation

Strictures in Crohn’s Disease, characterized by the narrowing of the intestinal lumen due to inflammation, fibrosis, or both, can lead to obstructive symptoms such as abdominal pain, bloating, and nausea [44]. Before considering dilation, it is important to perform magnetic resonance imaging to accurately determine the length and characteristics of the stricture. DAE can directly approach stenoses that are fibrotic, less than 5 cm, and without prestenotic dilation, allowing for endoscopic dilation [45]. This procedure involves the use of a balloon which is guided to the site of the stricture, and then expanded to widen the narrowed area [46]. Dilation can alleviate obstructive symptoms and restore bowel patency, improving the quality of life for patients with symptomatic strictures [47]. DAE has been shown to be an effective and relatively safe therapeutic option; a pooled analysis of individual data from 1463 patients revealed a technical success rate of 89.1%, clinical efficacy in 80% of patients, and a major complication rate of 2.8%, with symptomatic recurrence observed in 75% over a mean follow-up period of 24 months. A stricture length of 5 cm or less was correlated with a successful result without the need for surgery [48]. Further studies including metanalysis corroborate the efficacy and safety of endoscopic dilation for small-bowel Crohn’s Disease strictures, emphasizing its role in delaying surgical interventions and improving patient outcomes in the short term; however, up to two-thirds of patients need re-dilation or surgery [48,49]. 

### 6.2. Removal of Foreign Bodies

In the context of Crohn’s Disease, foreign bodies typically refer to undigested food particles or medication bezoars that can accumulate in areas of the intestine narrowed by strictures [50,51]. DAE allows for the direct visualization and removal of these foreign bodies, which can alleviate obstructive symptoms and prevent the progression to more severe complications, such as bowel obstruction or perforation [52]. DAE is also a feasible, relatively safe, and effective method to remove retained video capsule endoscopes. A recent systematic review demonstrated that the pooled successful retrieval rate using DBE was 86.5%, with a higher success for capsules retained in the jejunum or higher in the small bowel (100% retrieval success rate), and lower for those in the ileum (success rate of 74.1%). Successful capsule retrieval significantly reduced the need for subsequent surgeries. Only 7.2% of successful retrievals required surgery compared to 38.5% in unsuccessful cases, highlighting the benefit of effective DBE use [53].

### 6.3. Treatment of Bleeding Lesions

Bleeding in the small intestine can originate from disease-associated lesions and anastomotic ulcers, potentially causing significant blood loss and leading to anemia [54]. DAE enables the precise localization and treatment of these bleeding lesions, employing techniques such as argon plasma coagulation (APC) [55], endoscopic clipping [56], or the injection of hemostatic agents [47].

These endoscopic treatments can effectively manage and control bleeding, reducing the need for transfusions, further diagnostic testing, or surgical intervention [57]. The choice of technique is influenced by the lesion’s location, severity of bleeding, presence of associated complications (e.g., strictures), and the patient’s overall condition [58].

In cases where the bleeding is severe, a prior CT angiography may be relevant. CT angiography can help identify the precise bleeding site and assess the vascular anatomy, which is crucial for planning an appropriate treatment [59]. Some severe cases of bleeding can be more effectively managed with intravascular coiling, a minimally invasive procedure performed by interventional radiologists. Intravascular coiling involves the placement of coils to occlude the bleeding vessel, providing rapid hemostasis and minimizing the risk of recurrent bleeding [60]. 

For cases of severely bleeding ulcers, an endovascular radiological approach may also be preferable. This approach allows for the targeted delivery of embolic agents or coiling directly to the bleeding site, offering an alternative to endoscopic treatments when bleeding is not controlled or accessible via DAE [61,62]. The integration of endovascular techniques in the management of small-intestinal bleeding underscores the importance of a multidisciplinary approach, combining the expertise of gastroenterologists, interventional radiologists, and surgeons to optimize patient outcomes.

### 6.4. Steroid Injection

Endoscopic steroid injections have been studied as a treatment for Crohn’s Disease, particularly focusing on strictures and inflammation management [63]. Singh et al. highlight the use of intramural steroid injections in conjunction with endoscopic dilation, presenting a promising approach to managing CD-related strictures, indicating high success rates and suggesting the potential for reduced fibrosis and improved clinical outcomes [64]. Alesandra Lavy and colleague affirm the beneficial outcomes of steroid injections in CD strictures, pointing towards improved stricture management [65]. Di Nardo et al. contribute to this body of evidence with a prospective, randomized, double-blind, controlled trial focusing on pediatric CD patients, which underscores a steroid injection following endoscopic balloon dilation, providing evidence that it is a successful approach for decreasing the need for both redilation and surgery [66]. In contrast, a controlled trial by East et al. raised doubts about its safety and efficacy: patients receiving steroid injections showed a higher incidence of the need for repeat procedures and a shorter time to recurrence of the stricture [67]. In conclusion, there is currently insufficient evidence to support routine use in clinical practice without a large-scale controlled trial, and it should be noted that this approach is still considered experimental to date [68]. 

## 7. Impact on Disease Management and Patient Outcomes

The incorporation of DAE into the management of Crohn’s Disease has had a profound impact on disease strategy and patient outcomes. The ability to perform targeted interventions for complications that would otherwise require surgery has led to a reduction in surgical intervention rates [39]. This shift towards less invasive management options can have profound implications for patients, including reduced morbidity associated with surgery, the preservation of bowel length (crucial in preventing short bowel syndrome in a disease prone to multiple interventions over time), and decreased recovery times [69]. Furthermore, avoiding surgery can significantly impact the patient’s overall health trajectory, reducing the risks of post-operative complications and the potential for subsequent surgeries. Double-balloon enteroscopy (DBE) has been shown to have a substantial impact on the management and outcomes of CD by enabling a detailed examination and intervention within the small intestine [70]. A multicenter retrospective study highlighted its findings and management implications, showing DBE’s role in altering treatment strategies for many patients. The findings from DBE affected management in a high percentage of patients with documented and suspected CD (82% and 79%, respectively) [39]. 

## 8. Improvement in Symptoms and Quality of Life

DAE’s role in directly treating the complications of Crohn’s Disease contributes significantly to symptom relief. For instance, the dilation of strictures can immediately relieve obstructive symptoms such as abdominal pain, vomiting, and bloating, while the control of bleeding lesions can prevent anemia and associated fatigue, improving overall well-being [71]. Beyond the physical symptom relief, the minimally invasive nature of DAE, coupled with its efficacy in managing specific disease complications, contributes to an overall improvement in the quality of life. Patients may experience fewer disease flare-ups, reduced anxiety about their health, and greater engagement in social and professional activities, contributing to a more positive outlook on life despite living with a chronic condition [72].

## 9. Challenges and Limitations of DAE in Crohn’s Disease

While DAE offers significant advantages in diagnosing and managing Crohn’s Disease, it is not without its challenges and potential complications, especially perforation and bleeding. These factors must be carefully considered when opting for DAE as a diagnostic or therapeutic tool.

### 9.1. Risk of Perforation, Bleeding, and Pancreatitis

The major adverse events related to DAE are perforation, bleeding, and pancreatitis. One of the most serious complications in Crohn’s Disease is the risk of perforation. This risk is inherent to the nature of the procedure, which involves navigating and, sometimes, dilating the small intestine, an organ that may already be compromised by disease-related damage, including the thinning or weakening of the intestinal walls due to inflammation or fibrosis [73]. A recent systematic review found that the per-procedure perforation rate for diagnostic BAE in CD was 0.15%, which is comparable to the rate for diagnostic BAE across all indications. For therapeutic BAE in CD, the perforation rate was 1.74% per procedure. The majority of these therapeutic perforations, 86%, occurred as a result of stricture dilation [74]. Similarly, a multicenter survey in Portugal reported a perforation risk of 0.28% associated with DAE, indicating a relatively low but significant risk [75]. Perforation can lead to severe outcomes, requiring emergency surgical intervention and potentially leading to further complications such as infection or sepsis. DAE also carries a risk of inducing bleeding, especially when a procedure such as stricture dilation are performed. While bleeding is often less severe than perforation and can frequently be managed endoscopically, it nonetheless represents a significant risk, particularly in patients with Crohn’s Disease who may already be at an increased risk of bleeding due to their underlying condition. A retrospective study on 776 dilations performed on patients with Crohn’s Disease reported a risk of major bleeding, i.e., requiring blood transfusion, of 1% [76]. The estimated risk of acute pancreatitis is 0.3–1%; the proposed explanations for the onset of acute pancreatitis from enteroscopy include the rise in intraluminal pressure within the duodenum during the procedure, which causes duodenal fluids to flow back into the pancreatic duct [77].

The complication rate was evaluated in a large retrospective multicenter US study, which aimed to assess the safety, diagnostic, and therapeutic yields of DAE over a five-year period. Over 1787 instances of DAE, only 0.9% encountered complications, including two perforations (0.1%), six cases with bleeding (0.3%), and one episode of pancreatitis (0.1%) [78]. Similar results emerged from a large cohort study that examined 3894 cases, revealing an overall complication rate of approximately 1%, with pancreatitis as the most frequent complication in diagnostic exams [79].

### 9.2. Sedation Risks

DAE must be performed under deep sedation or general anesthesia, which carries inherent risks, especially in patients with compromised health [80]. A large German registry indicated that 0.5% of complications during DAE were associated with sedation. While DAE can be safely performed on an outpatient basis, it is recommended that we conduct the procedure as an inpatient procedure with extended monitoring for patients with significant comorbidities [81]. Prolonged sedation can lead to respiratory depression, hypotension, and other anesthesia-related complications. Studies have shown that the length of the procedure can increase the risk of these complications. For instance, a study examining the safety of prolonged endoscopic procedures under sedation found that an extended procedure time was associated with a higher incidence of sedation-related adverse events, including hypoxia and hypotension [82].

### 9.3. Skill and Experience Requirements

The successful and safe performance of DAE requires a high degree of skill and expertise. This is because DAE procedures involve complex techniques for navigating the small intestine, a challenging and lengthy part of the gastrointestinal tract. The ability to effectively manage the equipment, recognize and navigate around potential complications, and perform therapeutic interventions requires extensive training and experience. The outcomes of DAE, including both its diagnostic yield and the success rate of therapeutic interventions, are closely linked to the operator’s experience. Studies have shown that higher volumes of procedures are associated with improved outcomes and reduced complication rates. This necessitates a concentrated effort to train gastroenterologists in these techniques, which may not be available in all healthcare settings, potentially limiting access to DAE for some patients.

### 9.4. Addressing the Challenges

The challenges and limitations of DAE underscore the need for careful patient selection, thorough pre-procedural planning, and the judicious use of DAE by skilled and experienced practitioners. Strategies to mitigate these risks include the use of pre-procedural imaging to assess the feasibility and safety of DAE, ongoing training and education for endoscopists, and the development of guidelines to standardize the procedure and manage complications effectively [83,84].

In conclusion, while DAE represents a significant advancement in the management of Crohn’s Disease, its technical challenges and potential complications require a careful and considered approach. Through specialized training, experience, and the adherence to best practices, the risks associated with DAE can be minimized, maximizing its benefits for patients with Crohn’s Disease [85].

## 10. Conclusions

DAE has solidified its role as a pivotal tool in the management of Crohn’s Disease by providing comprehensive insights into the small intestine and offering both diagnostic and therapeutic capabilities. As the technology and techniques of DAE continue to evolve, the potential to further improve patient care and outcomes in Crohn’s Disease remains significant. Ongoing research and development are vital to maximizing the utility of DAE, ensuring patient safety, and expanding its therapeutic applications. The continuous improvement of DAE technology and techniques is essential in order to enhance its safety profile, reduce procedural risks, and expand its therapeutic capabilities.

## Data Availability

Not applicable.
